# Development of a welfare assessment protocol for dromedary camels during road transport

**DOI:** 10.3389/fvets.2026.1868838

**Published:** 2026-07-10

**Authors:** Barbara Padalino, Asim Faraz, Patrick Meyer-Glitza, Anam Zohra, Faizan Saleem, Hassan Qadir Buzdar, Nasir Ali Tauqir, Naod Thomas Masebo

**Affiliations:** 1 Faculty of Science and Engineering, Southern Cross University, Lismore, NSW, Australia; 2 Department of Agricultural and Food Sciences, University of Bologna, Bologna, Italy; 3 Department of Livestock and Poultry Production, Bahauddin Zakariya University, Multan, Pakistan; 4 Animals’ Angels, Frankfurt, Germany; 5 Departement of Veterinary and Animal Sciences, Ghazi University, Dera Ghazi Khan, Punjab, Pakistan

**Keywords:** indicators, journey, loading, loading density, unloading, vehicle

## Abstract

Transporting dromedary camels, like other farm animals, may be stressful and impair their welfare and health. The lack of standardized practices regarding loading and unloading, vehicle design and size, journey duration, and space allowance complicates camel transportation, leading to greater welfare compromise and risk. The study aimed to develop a protocol for assessing the welfare of dromedary camels during road transportation. A group of researchers and experts (*n* = 10) conducted an extensive literature search and frequent meetings, during which several unstructured expert knowledge elicitation (EKE) sessions were conducted to develop the protocol. The literature search was conducted in Scopus and Web of Science databases; additionally, a snowball search was conducted to locate relevant literature. Overall, 24 articles from the databases, 10 initial articles, and 20 articles from the snowball search were used. Due to limited research on the transportation of dromedary camels, the authors primarily considered studies on the welfare assessment of other species during transport, such as horses, pigs, and ruminants. Accordingly, the checklists were prepared to assess the welfare of dromedary camels at five stages during transportation: before loading (24 indicators), after loading (18 indicators), during the journey (14 indicators), upon arrival (24 indicators), and 1 h after arrival (22 indicators). Additionally, a checklist was prepared to assess the loading and unloading process (28 indicators), the journey, and the vehicle (17 indicators). This is the first protocol to assess the welfare of dromedary camels during road transportation. It uses non-invasive methods and practically applicable welfare measures suitable for field conditions. However, it needs to be applied to refine and validate the proposed animal-based and environmental welfare indicators.

## 1 Introduction

Transportation is a major part of the current livestock industry, but it may greatly affect animal health, welfare, carcass, and meat quality (1–3). Changes in livestock farming have increased the transportation of animals due to the rising demand for meat, milk, and international trade (4–6). Most farm animals typically experience transportation at least once in their lifetime (7). The livestock transportation process involves mustering, preparation, loading, transport, and unloading (1, 8). It is well known that loading and unloading processes, loading density, microclimate conditions in the vehicle, journey duration, mixing different animals, the type of animal transported, food and water deprivation are welfare hazards (1, 9–12). Consequently, animal welfare assessment conducted during transport should be multidimensional and cover all phases of the journey (13). The assessment should use both animal-based measures (ABMs) and resource-based measures (14–16). Hence, the assessment should consider the animal, the journey conditions, journey length, the transport vehicle, and the loading and unloading processes (9, 16, 17).

The farming system of the dromedary camel is, in the majority of cases, extensive, although there is a trend toward intensification (18, 19). Therefore, animals are rarely confined to one space. Consequently, during the transportation of dromedary camels, attention should be given to the farming system they are coming from; the knowledge and experience of handlers, drivers, and stock people are detrimental (20). Dromedary camels are farm animals found in arid regions of the world, in harsh environments characterized by high temperatures, high solar radiation, and a shortage of water and food (18, 21). However, it has been reported that there is an emergence of intensive and peri-urban camel farming (19, 22). Researchers are increasingly interested in dromedary camels because of their physiological adaptations that enable them to survive in harsh environments and extreme weather. Additionally, there has been an increase in demand for camel milk, and they are being considered as potential future farm animals (18, 23, 24). Consequently, the number of movements of dromedary camels across the globe has increased. Similar to other farm animals, transporting dromedary camels is stressful and negatively impacts their welfare, as demonstrated by several studies reporting the effects of transportation on physiological, hormonal, biochemical, and hematological parameters (25–29). It was reported that the level of cortisol, Packed cell volume (PCV), neutrophils (27), hematocrit, haemolysis, cortisol, glucose, oxidative stress parameters (30), lactate, malondialdehyde, and catalases increased after transportation (25). Even short road transportation for up to 5 hrs alters the physiological parameters of dromedary camels, impairing their welfare (29). Dromedary camels are transported to different locations for different purposes, such as meat production at young ages, dairy production, cosmetic and sporting events like racing (31, 32). The transport conditions, the infrastructure, equipment, and animal being transported determine the effect of transportation on the animals (20). Extensively reared animals can pose a different risk since their management system is different, and the response of the animal may be different and diverse to transportation stress (20).

In other farmed animals, several protocols to assess welfare on the farm and during transport have been developed, and there are clear indications for evaluating fitness for transport before loading and welfare status on arrival (33–37). These protocols provide standardized indicators based on animal-based, resource-based, and management measures. Recently, protocols have been developed to assess the welfare of dromedary camels in extensive, semi-intensive, and intensive systems (38, 39); however, no protocol exists to evaluate the welfare of dromedary camels during road transport. Moreover, in many camel-rearing regions, camel transportation is mostly traditional and unregulated without any standard practices (31, 40). Although standards from the World Organization for Animal Health exist (41), they are not widely implemented. The lack of standardized practices regarding loading and unloading, vehicle design and size, journey duration, loading density, and space allowance complicates camel transportation, leading to greater welfare compromise and risk (31, 40). Therefore, this study aimed to develop a protocol for assessing the welfare of dromedary camels during road transportation.

## 2 Materials and methods

### 2.1 Protocol development and selection of indicators

The first and last authors (BP and NTM) invited several (*n* = 20) colleagues with expertise in camel science to take part in this study, to balance different backgrounds, namely academia, NGOs and stakeholders. In the end, a group of 10, namely two members of an NGO, one stakeholder (i.e., one veterinarian working in a camel practice), two animal scientists, 4 veterinarians working in academia with a strong focus on camel science, and 1 veterinarian specialist in animal welfare, worked together on this protocol. As a first step, the first and last authors conducted an extensive literature search to select possible papers from which to screen valid animal-based and environmental-based indicators to include in the protocol (42). The initial indicators were taken from the two papers describing the protocols used to assess the welfare of camels at farms in different husbandry systems (38, 39). Moreover, eight manuscripts assessing the welfare of livestock at different stages of transportation, including horses, sheep, and pigs, and the protocols used in these studies were included (35, 36, 43–48).

**Figure 1:**
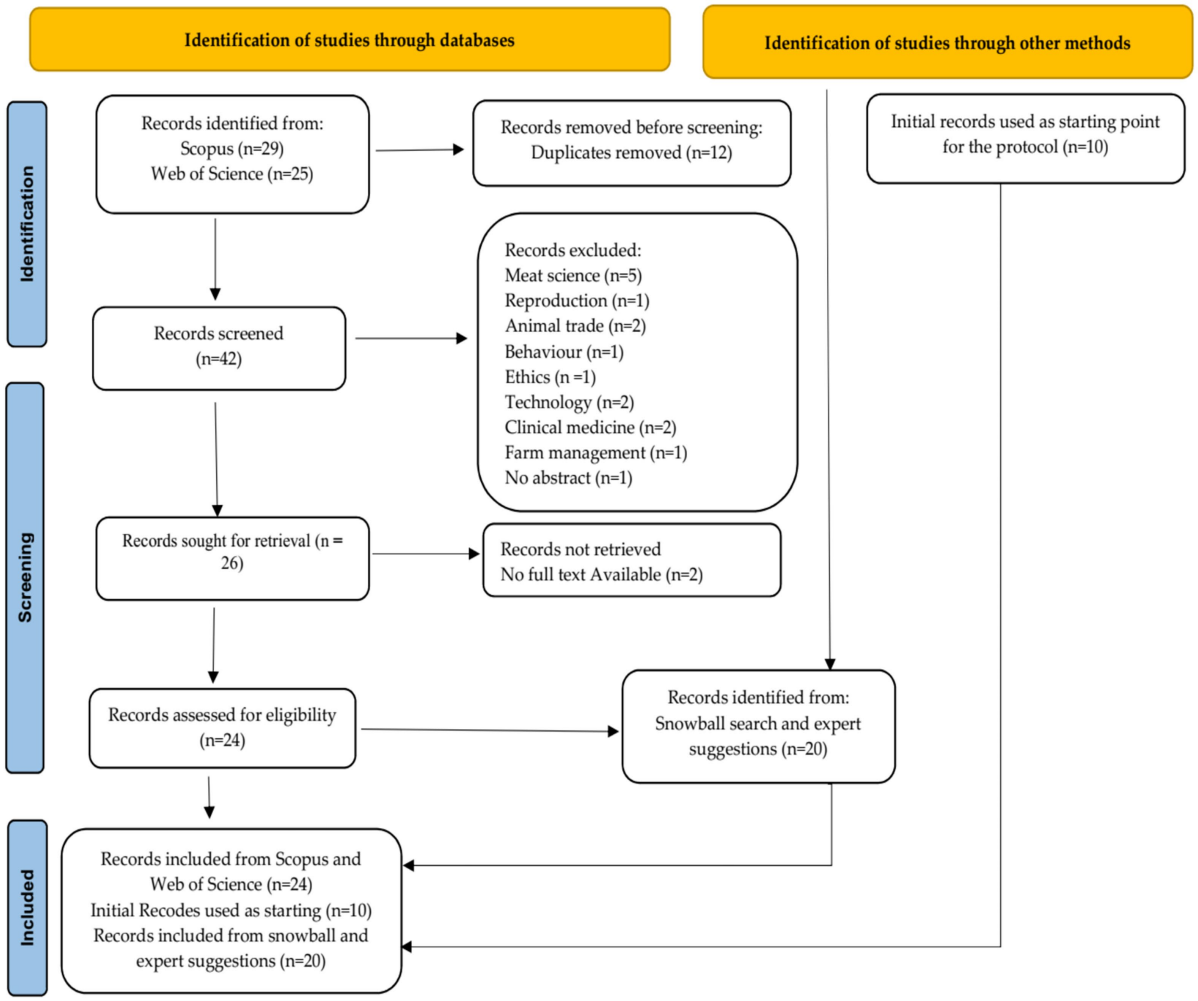
Selection procedure and total number of records used to develop the protocol. The figure also reports the number of excluded records and the exclusion criteria.

The scientific literature search was conducted using Scopus and Web of Science databases. The authors discussed the search strings, and numerous trials were carried out to determine which strings best suited the search’s objectives. The final search strings used in Scopus contains the following word combinations: (“dromedary camel*” OR horse* OR cattle OR pig* OR livestock) AND (“animal transport” OR “transportation of animals”) AND (“welfare assessment” OR “animal welfare”) AND (indicator* OR protocol* OR stress* OR behavior* OR physiological) and in Web of science, the following was used TS = (“welfare assessment” OR “animal welfare”) AND TS = (indicator* OR protocol* OR stress* OR behavior* OR physiological). It is noteworthy that the literature review was confined to English-language publications in the last 10 years. In total, 43 peer-reviewed articles were retained after removing the duplicates. Then, the first and senior authors (BP, NTM) screened the results by reading the titles, keywords, and abstracts for relevance after agreeing on the inclusion and exclusion criteria (Table 1). Accordingly, 16 records were excluded. In addition, a snowball search was conducted to identify relevant literature for the protocol preparation (Figure 1). Due to the scarcity of literature regarding the welfare assessment of dromedary camels during transportation, the research team focused on peer-reviewed studies concerning the development and implementation of welfare assessment protocols for other species, including horses and ruminants.

**Table 1:** Inclusion and exclusion criteria applied to screened records.

Inclusion criteria	Exclusion criteria
Studies and reviews on the welfare assessment of farm animals during transport, such as horses, pigs, cattle, and sheep	Studies focusing on the effects of transport on meat quality
Studies and reviews on the fitness of animals for transport (horses, cattle, pigs)	Studies on physiology, reproduction, behavior, ethics, and clinical cases not related to transport
Studies focusing on the welfare assessment of animals during transport (for example, welfare assessment on arrival in horses)	Studies focusing on international animal trade
Studies on the effects of transport on dromedary camels	Studies on farm management and husbandry technologies
Studies on the welfare assessment of farm animals	

**Figure 2:**
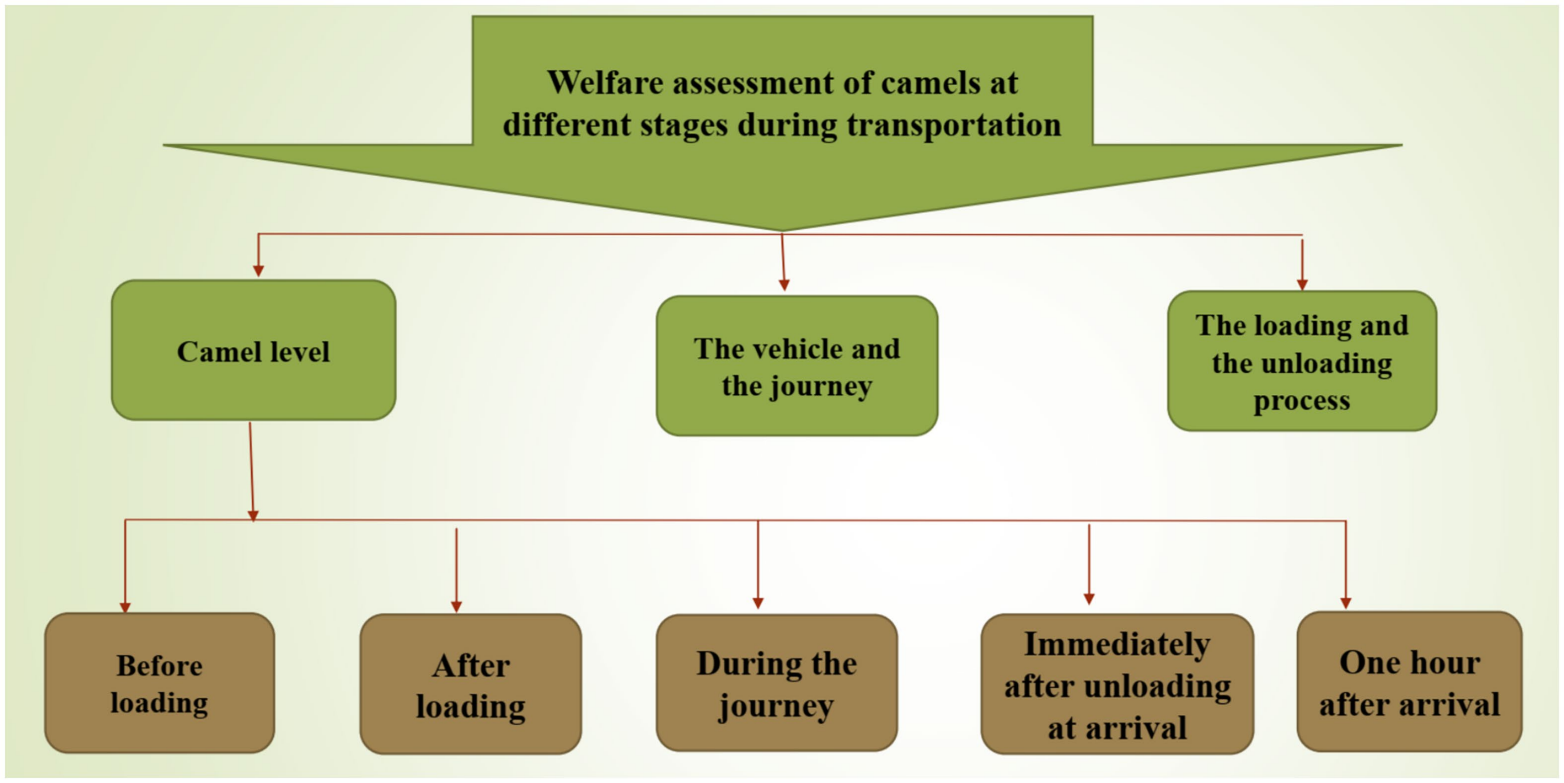
Illustrating the welfare assessment of camels at different stages during transportation.

Then the first and last authors organized online meetings (n = 5), during which several unstructured expert knowledge elicitations (EKEs) were conducted to select measurement indicators and develop the protocol (42). During the first meeting between the authors and the other experts, it was decided to include only non-invasive measure indicators in the protocol checklists, so all parameters which required invasive methodology, namely blood sampling and other invasive veterinary procedures, were excluded. The development of a non-invasive protocol to assess animal welfare during transport is paramount to improving the transportation process by investigating the factors that contribute to negative welfare consequences during transport (17). Based on the literature review and expert consensus, the welfare assessment indicators for dromedary camels were defined to include both animal-based and resource-based measurements (2, 9, 13, 15, 16, 35, 49). During the second meeting, a long discussion on when to perform the assessment was conducted. Consequently, five different checklists were prepared to assess the welfare of dromedary camels at the animal level at five stages during transportation: before loading, after loading, during the journey, upon arrival, and 1 h after arrival. Additionally, an *ad hoc* checklist was prepared to assess the loading and unloading process, the journey, and the vehicle (Figure 2).

After having agreed on the time points, the first and the last author worked on the possible checklists. The checklists were developed based on their experience in animal transportation, camel behavior, and handling, as well as on existing welfare protocols for camels under different management systems (38, 39) and for other livestock species during transport (35, 43, 45–48). During the last third and the fourth meetings, the indicators to retain in each checklist were discussed and finalized. To reach consensus, online polls were conducted asking the members to rank from 1 to 5 the reliability and the feasibility of each indicator in each of the 5 checklists. Only indicators which were scored highly reliable and feasible by at least 7/10 experts were retained as suggested in the literature (49, 50). Statistical analysis was not conducted, as consensus was always reached during the meetings. It was also agreed that the work flow to collect the data during the assessment had to follow what was reported in the literature (38, 39, 45). The last ad fifth meeting was used to score all indicators. The scores were decided based on the literature, namely on a scale of 0–2, to give all indicators the same weight (51).

**Figure 3:**
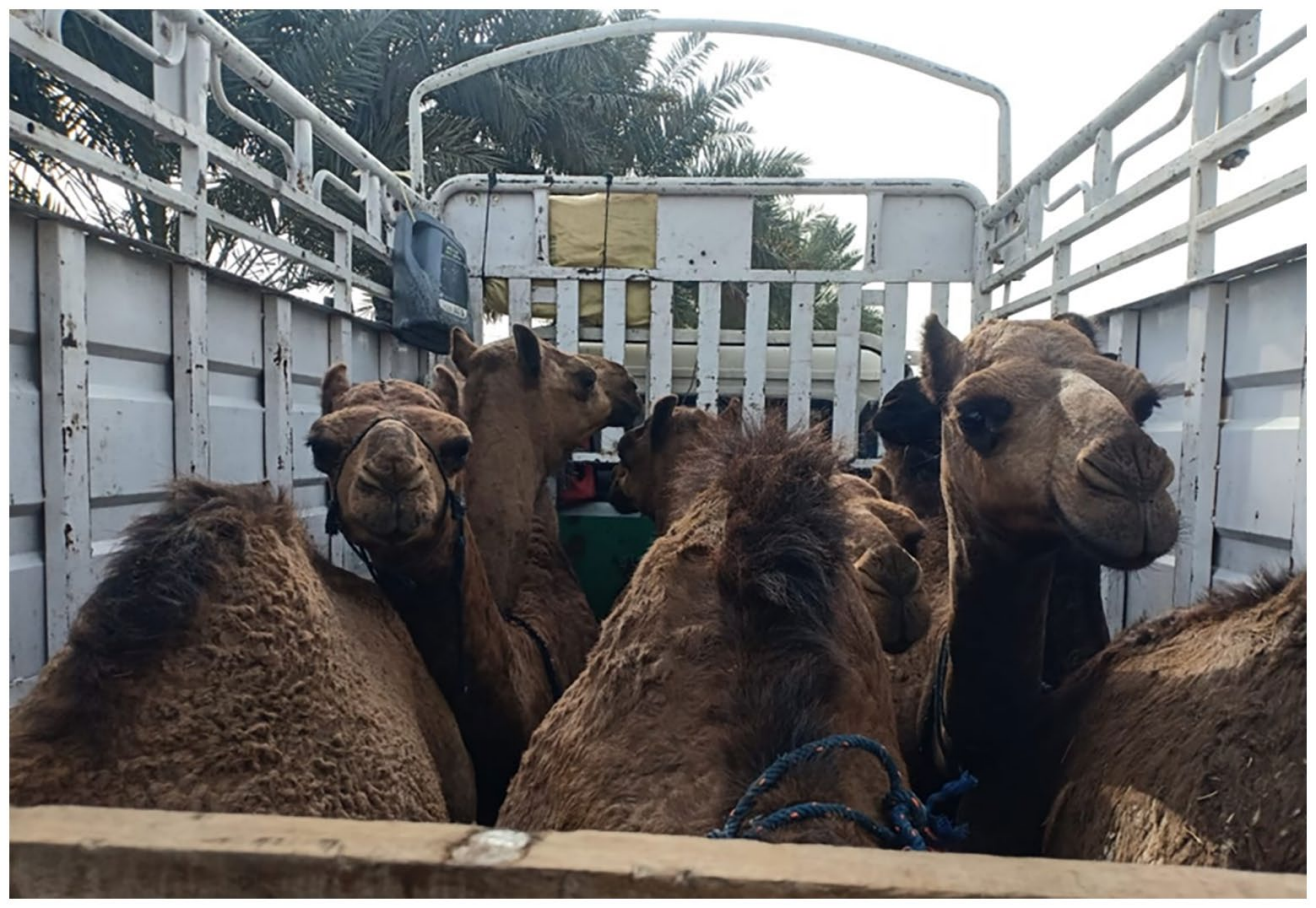
Picture of the six male dromedary camels used to pilot the protocol during the first journey.

**Figure 4:**
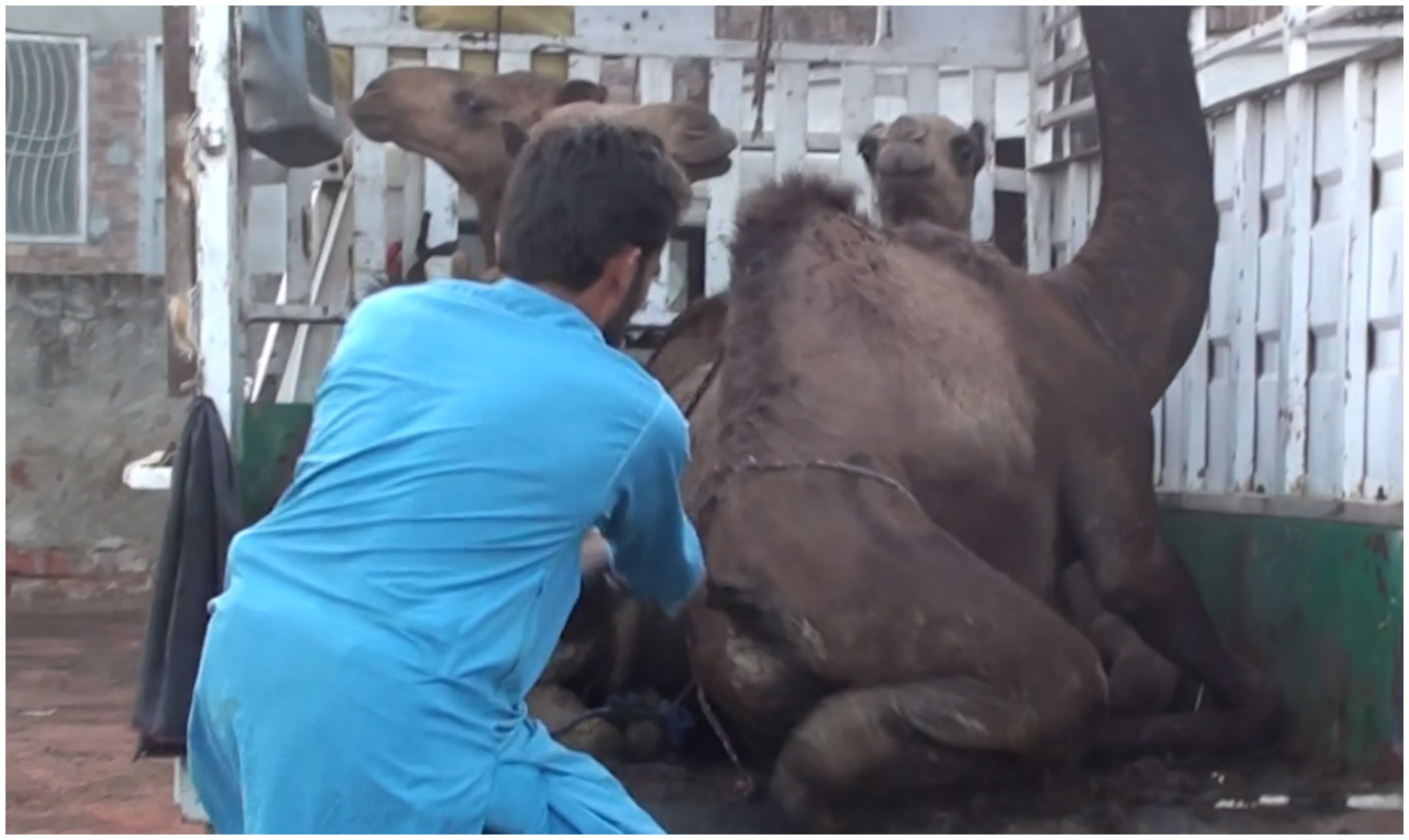
Manual unloading of camel (all camels were pulled down manually with excessive force at the end of that journey).

The checklists were then piloted, during three journeys in the southern Punjab province of Pakistan. In particular, in the first journey, 6/6 young males aged about 8 months were assessed, in the second journey, 7 (5 young males, 1 young female, and 1 adult lactating female) out of a group of 22 camels were assessed, and 6/6 males aged about 1 year were assessed. Camels were collected from pastoralists by middlemen and traders and taken to collection centers. The camels were loaded onto vehicles and transported to slaughterhouses. This journey usually took place in the evening and lasted an average of 12 h. Based on our preliminary observations, we removed some indicators that were not feasible and difficult to apply from the checklists. For instance, measuring the body temperature and heart rate of the camel were removed as judged not feasible for field conditions. One of the problems is the limited space allowance (an average of 0.73 m^2^) (Figure 3), which makes it impossible for the assessor to approach each camel after loading and be able to record those parameters using a stethoscope or a thermometer. At unloading, the camels were exhausted, unable to stand immediately, but stress-related vocalizations were observed, suggesting including vocalizations as one of the parameters, and the fact that the same camels were able to walk properly at the check 1 h after the journey, strengthened the idea of the double check after transport. As during both loading and unloading, rough handling (e.g., pulling tails for unloading the animals) was observed, this indicator was added in the checklists (Figure 4).

One hour after loading, swollen joint (8.3%), skin disorder (15.4%), injuries (8.3%), lameness (8.3%), and nasal discharges (8.3%) were noticed. No mortality was recorded in all three journeys. However, it was decided to keep the mortality rate and the number of downers in the checklists. After piloting, it was decided to keep the checklist during the journey at the group level, as individual assessment was judged to be very time-consuming and not feasible during rest stops.

## 3 Results

The protocol has different sub-sections containing checklists used to assess the welfare of dromedary camels at different stages of transportation, including the assessment of the camel before loading, after loading, during the journey, upon arrival, and 1 h after arrival; plus, it has a section of the protocol to assess the loading and unloading process, the vehicle, and the journey.

### 3.1 Assessment of camels before loading

The first checklist at camel level must be filled before loading to evaluate the camel’s fitness for transport and determine whether the camel is suitable to be transported. Animals that are not fit for travel are most likely to suffer more negative welfare consequences than fit animals, and should not be loaded (13, 14, 52, 53). The assessment of welfare before loading includes the evaluation and recording of body condition score (BSC), presence of injury, presence of blood, clinical physical examinations such as respiratory rate, and, in addition, behavioral assessment should also be conducted (15, 16, 38, 39). The checklist for assessing dromedary camel welfare before loading and deciding whether every single animal is fit or not for transport is presented in Table 2. Although there are no literature or studies regarding the fitness of dromedary camels for transport, the authors and expert group extensively reviewed articles written for other farmed animals, such as cattle, small ruminants, pigs, and horses, and compiled a list of conditions that render dromedary camels unfit for transport (Table 3).

**Table 2:** Checklist to evaluate fitness for transport in dromedary camels before loading, agreed using EKEs.

Camel ID: ______Time: ______ Date: _________ ID of the Journey: ____________ Sex: ____ Age: _________ Breed: __________Physiological State: ____________BW: ___ Ambient temperature: ____________ Humidity: _______________				
No	Variable	Measurement scales /values	Score obtained	Remarks
1.	Respiratory rate (74)	Normal (8–16 bpm/min)	0	
		Abnormal (>16 bpm/min)	2	
2.	Body condition score (BSC) (75)	BCS = 3 (good body condition)	0	
		BSC = 2 or BSC = 4 (Moderate body condition)	1	
		BSC = 0–1 (cachexia) or BCS = 5 (obesity)	2	
3.	Dehydration	Normal	0	
		Abnormal (visible dehydration)	2	
4	Fatigue/exhaustion	No	0	
		Yes	2	
5.	Presence of blood	No	0	If yes, the location ____________
		Yes	2	
6.	Presence of injuries or cuts	No	0	If yes, the location ____________
		Yes	2	
7.	Presence of swollen joints	No	0	If yes, the location _________
		Yes	2	
8.	Presence of lameness	No	0	If yes, what grade:______
		Yes	2	
9.	Presence of skin disorders	No	0	
		Yes	2	
10.	Presence of respiratory disorders	No	0	
		Yes	2	
11.	Presence of discharge (nose, eye, vulva) (watery, bloody mucus, purulent, smelly, etc.)	No	0	Type and origin of the discharge:___________
		Yes	2	
12.	Presence of eye lesions	No	0	
		Yes	2	
13.	Presence of coughing	No	0	
		Yes	2	
14.	Presence of salivation	No	0	
		Yes	2	
15.	Presence of diarrhea	No	0	
		Yes	2	
16.	Presence of other health disorders	No	0	Specify: ____________
		Yes	2	
17.	Presence of rectal or uterine prolapse	No	0	
		Yes	2	
18.	Presence of pregnancy (female camel)	No	0	
		Yes	1	
19.	Recent calving (female camel)	No	0	
		Yes	1	
20.	Presence of pain-induced management practices (nose peg)	No	0	
		Yes	1	
21.	Demeanors	Bright, alert, responsive (BAR)	0	
		Nervous	1	
		Lethargic/ Exhausted	2	
22.	Stereotypes	No	0	
		Yes	2	
23.	Approach test	positive	0	
		negative	1	
		neutral	2	
24.	Is the camel in good fitness to be transported?	Yes	0	If no, why __________________
		No	2	

**Table 3:** List of conditions rendering dromedary camels unfit for transport.

Conditions	Descriptions
Severe lameness	The camel is unable to walk normally or bear weight in all four legs (76)
Severe injuries	The camel has a fracture or severe injury (15, 53)
Wounds	The camel has severe wounds/open, sizeable skin lesions and visible fresh blood (15, 58)
Eye lesions	The camel is blind in both eyes, or the camel has a severe lesion on the eye(s) (15)
Severe or visible dehydration	The camel has visible signs of dehydration, showing reduced skin elasticity (> 5 s), sunken eyes, and dry mucus membranes (15, 53)
Skin disorders	The camel has a severe skin infection (e.g., Fungus) (77, 78) (ref)
Fatigue/exhaustion	The camel is very weak and unable to stand (15, 53)
Illness	The camel has visible clinical signs such as high fever, severe diarrhea, and other visible illnesses (58)
Suffering from pain	The camel is exhibiting signs of pain and is not responding to the surrounding environment; it suffers from pain when moved (79)
Severe respiratory distress	The camel is breathing heavily, panting, labored breathing (80, 81)
Emaciation	The camel is very thin and has very poor body condition (BCS = 0–1) (15, 53, 75, 82)
Generalized nervous disorders	The camel is unable to maintain balance, and/or shows signs of nystagmus (15, 53, 83)
Prolapse	The camel has rectal or uterine prolapse (58, 84, 85)
Advanced pregnancy	The camel is in the advanced stage of pregnancy or close to parturition (last month of pregnancy) (15, 53)
Recent calving	The female camel gave birth recently (in the previous week) (15, 41, 53).
Newborn	The camel is newborn with an unhealed navel or less than 1 week old (14, 15, 41).

### 3.2 Welfare assessment of dromedary camels after loading

Table 4 shows the checklist to assess camel welfare after loading. After loading, camels are assessed to determine whether they have sustained any injuries, experienced stress, and to evaluate their positions within the truck. Therefore, assessment of camels is performed after loading while the camels are on the truck. The evaluation includes the presence of conditions, signs of disease, blood, injuries, aggressive behavior, and stereotypic behaviors.

**Table 4:** Checklist to evaluate the welfare of dromedary camels after loading agreed using EKEs.

Camel ID: ______Time: ______ Date: _________ ID of the Journey: ____________ Sex: ____ Age: _________ Breed: __________Physiological State: ____________BW:___ Ambient temperature: ____________ Humidity: ________________				
No	Variable	Measurement scales /values	Score obtained	Remarks
1	Position of camels on the truck during the journey	Sternal position without restraint	0	Other: __________
		Sternal position with restraint	1	
		Standing	2	
2.	Are the camels restrained in the truck?	No	0	If yes, how? ____________
		Yes	2	
3.	Presence of blood	No	0	
		Yes	2	
4.	Presence of injuries or cuts	No	0	If yes, the location ____________
		Yes	2	
5.	Presence of swollen joints	No	0	
		Yes	2	
6.	Presence of skin disorders	No	0	
		Yes	2	
7.	Presence of discharge (nose, eye, vulva) (watery, bloody mucus, purulent, smelly, etc.)	No	0	Type and origin of the discharge:___________
		Yes	2	
8.	Presence of eye lesions	No	0	
		Yes	2	
9.	Presence of coughing	No	0	
		Yes	2	
10.	Presence of salivation	No	0	
		Yes	2	
11.	Presence of diarrhea	No	0	
		Yes	2	
12.	Presence of respiratory disorders	No	0	
		Yes	2	
13.	Presence of other health disorders	No	0	If yes, specify: ____________
		Yes	2	
14.	Sweating	No	0	
		Yes	2	
15.	Demeanors	Bright, alert, responsive (BAR)	0	
		Nervous	1	
		Lethargic/Exhausted	2	
16.	Stereotypes	No	0	
		Yes	2	
17.	Do camels have enough space?	Yes	0	How much space does each camel have: ________ m ^2^
		No	2	
18.	Loading density	Kg/m^2^		

### 3.3 Assessment of dromedary camels during the journey

The checklist to assess the camels during the journey is presented in Table 5. During the journey, the camels must be assessed when the vehicle is stopped, possibly in the middle of the journey when the driver stops for rest, or before the journey is completed. This checklist should be filled in an opportunistic way, as no extra stop is requested; the welfare assessments stop will affect the welfare of the animals (15, 53). It is difficult and not feasible to assess individual camels while they are on the vehicle. Therefore, the authors and expert groups decided that the assessments should be conducted on a group basis. The evaluation includes the general behavior exhibited by the camels, the presence of vocalizations, whether the camels are ruminating, whether there are visible injuries or cuts, and the presence of stereotypic behaviors.

**Table 5:** Checklist to evaluate the welfare of dromedary camels during the journey, agreed using EKEs.

Date:________________ Time:____________ ID of the Journey: _____________ Number of camels: _________ Ambient temperature: ____________Humidity: ________________					
No	Variables	Number of camels	Criteria	Score obtained	Remarks
1.	Proportion of camels in sternal position without restraint		0–33%	2	
			34–66%	1	
			67–100%	0	
2.	Proportion of camels in sternal position with restraint		0–33%	0	
			34–66%	1	
			67–100%	2	
3.	Proportion of camels in standing position		0–33%	0	
			34–66%	1	
			67–100%	2	
4.	Proportion of camels vocalizing		0–33%	0	
			34–66%	1	
			67–100%	2	
5.	Proportion of camels ruminating		0–33%	2	
			34–66%	1	
			67–100%	0	
6.	Proportion of camels tied or restrained?		0–33%	0	
			34–66%	1	
			67–100%	2	
7.	Proportion of camels showing stereotypes		0–33%	0	
			34–66%	1	
			67–100%	2	
8.	Proportion of camels showing aggression toward other camels		0–33%	0	
			34–66%	1	
			67–100%	2	
9.	Proportion of camels with injuries or cuts		0–33%	0	The location of injuries or cuts ____________
			34–66%	1	
			67–100%	2	
10.	Proportion of camels with blood		0–33%	0	
			34–66%	1	
			67–100%	2	
11.	Proportion of camels showing health disorders		0–33%	0	Specify: ____________
			34–66%	1	
			67–100%	2	
12.	Proportion of camels sweating		0–33%	0	
			34–66%	1	
			67–100%	2	
13.	Proportion of camels salivating		0–33%	0	
			34–66%	1	
			67–100%	2	
14.	Proportion of camels showing discharge (nose, eye, vulva) (watery, bloody mucus, purulent, smelly, etc.)		0–33%	0	Type and origin of the discharge: _________
			34–66%	1	
			67–100%	2	

### 3.4 Welfare assessment of dromedary camels immediately after arrival

Immediately after arrival, the welfare condition of each camel is assessed. Each camel is examined individually for injuries, blood, and visible symptoms of disease. Their body condition score should also be assessed. Furthermore, whether there is any mortality or not (dead on arrival) should also be recorded at the destination. The checklist developed to assess the welfare of camels upon arrival is presented in Table 6.

**Table 6:** Checklist to evaluate the welfare of dromedary camels immediately after unloading upon arrival, agreed upon using EKEs.

Camel ID: ______Time: ______ Date: _________ ID of the Journey: ____________ Sex: ____ Age: _________ Breed: __________Physiological State: ____________BW:___ Ambient temperature: ____________Humidity: ________________				
No	Variable	Measurement scales /values	Score obtained	Remarks
1.	Respiratory rate (74)	Normal (8–16 bpm/min)	0	
		Abnormal (>16 bpm/min)	2	
2.	Body condition score (BSC)	BCS = 3 (good body condition)	0	
		BSC = 2 or BSC = 4 (Moderate body condition)	1	
		BSC = 0–1 or BSC = 5 (cachexia or obesity)	2	
3.	Dehydration	Normal	0	
		Abnormal (visible dehydration)	2	
4.	Fatigue/exhaustion	No	0	
		Yes	2	
4.	Presence of blood	No	0	
		Yes	2	
5.	Presence of injuries or cuts	No	0	If yes, the location __________
		Yes	2	
6.	Presence of swollen joints	No	0	
		Yes	2	
7.	Presence of lameness	No	0	If yes, what grade:______
		Yes	2	
8.	Presence of skin disorders	No	0	
		Yes	2	
9.	Presence of discharge (nose, eye, vulva) (watery, bloody mucus, purulent, smelly, etc.)	No	0	Type and origin of the discharge:___________
		Yes	2	
10.	Presence of eye lesions	No	0	
		Yes	2	
11.	Presence of coughing	No	0	
		Yes	2	
12.	Presence of salivation	No	0	
		Yes	2	
13.	Presence of diarrhea	No	0	
		Yes	1	
14.	Presence of sweating	No	0	If yes, how many _______
		Yes	2	
15.	Presence of respiratory disorders	No	0	
		Yes	1	
16.	Presence of rectal or uterine prolapse	No	0	
		Yes	2	
17.	Presence of other health disorders	No	0	Specify: __________
		Yes	2	
18.	Demeanors	Bright, alert, responsive (BAR)	0	
		Nervous	1	
		Lethargic/Exhausted	2	
19.	Stereotypes	No	0	
		Yes	1	
20.	Approaching test (51)	Positive	0	
		Neutral	1	
		Negative	2	
21.	Availability of water upon arrival	Yes	0	
		No	2	
22.	Availability of food upon arrival	Yes	0	
		No	2	
23.	If there is any downer at arrival?	No	0	If yes, how many _______
		Yes	2	
24.	If there is any mortality at arrival	No	0	If yes, how many _______
		Yes	2	

### 3.5 Welfare assessment of dromedary camels after 1 h of arrival

The welfare of camels after 1 h of resting should be assessed. Physiological parameters such as body temperature and respiratory rate should be recorded. In addition to behavioral assessment, the presence of injury, the presence of blood, the presence of respiratory signs such as coughing, and the presence of any abnormality should be checked. The checklist to assess camels 1 h after arrival is presented in Table 7.

**Table 7:** Checklist to evaluate the welfare of dromedary camels after 1 h of arrival as agreed using EKEs.

Camel ID: ______Time: ______ Date: _________ ID of the Journey: ____________ Sex: ____ Age: _________ Breed: __________ Physiological State: ____________BW: ___ Ambient temperature: ____________Humidity: ________________				
No	Variable	Measurement scales / values	Score obtained	Remarks
1.	Respiratory rate (74)	Normal (8–16 bpm/min)	0	
		Abnormal (>16 bpm/min)	1	
2.	Dehydration	Normal	0	
		Abnormal (visible dehydration)	2	
3.	Fatigue/exhaustion	No	0	
		Yes	2	
3.	Presence of blood	No	0	
		Yes	2	
4.	Presence of injuries or cuts	No	0	If yes, the location _________
		Yes	2	
5.	Presence of swollen joints	No	0	
		Yes	2	
6.	Presence of lameness	No	0	
		Yes	2	
7.	Presence of skin disorders	No	0	
		Yes	2	
8.	Presence of discharge (nose, eye, vulva) (watery, bloody mucus, purulent, smelly, etc.)	No	0	Type and origin of discharge: ___________
		Yes	2	
9.	Presence of eye lesions	No	0	
		Yes	2	
10.	Presence of coughing	No	0	
		Yes	2	
11.	Presence of salivation	No	0	
		Yes	2	
12.	Presence of diarrhea	No	0	
		Yes	2	
13.	Presence of sweating	No	0	
		Yes	2	
14.	Presence of respiratory disorders	No	0	
		Yes	2	
15.	Presence of other health disorders	No	0	Specify: ___________
		Yes	2	
16.	Presence of rectal or uterine prolapse	No	0	
		Yes	2	
17.	Demeanors	Bright, alert, responsive (BAR)	0	
		Nervous	1	
		Lethargic/Exhausted	2	
18.	Stereotypes	No	0	
		Yes	2	
19.	Approaching test (51)	Positive	0	
		Neutral	1	
		Negative	2	
20.	Availability of water	Yes	0	
		No	2	
21.	Availability of food	Yes	0	
		No	2	
22.	If there is any mortality after 1 h of arrival	No	0	If yes, how many _______
		Yes	2	

### 3.6 Assessment of the vehicle and the journey

In addition to assessing the camel, resources such as the means of transport (e.g., vehicle/truck) and the journey conditions are also evaluated. Therefore, the duration of the journey, distance traveled, stops during the journey, and the type of roads are recorded. The checklist developed to evaluate the vehicle and journey is presented in Table 8.

**Table 8:** Checklist to evaluate the condition of the vehicle and journey agreed using EKEs.

Date: __________ ID of the Journey: __________________Time: _____________ Ambient temperature: _________________ Humidity: ___________________				
No	Variables	Measurement scale/values	Score obtained	Remarks
1.	Description of the vehicle/Type of the vehicle			
2.	Dimensions of the truck cargo bed	Width in meters		
		Length in meters		
3.	Presence of a roof on the vehicle?	Yes	0	
		No	2	
4.	Presence of side protection in the vehicle? (to protect wind/sand)	Yes	0	
		No	2	
5.	Presence of a ramp in the vehicle	Yes	0	If Yes, Specify: ____________
		No, but loading ramp at the destinations (at loading and unloading)	1	
		No	2	
6.	Presence of protective side rails in the ramp?	Yes	0	
		No	2	
7.	Ventilation	Artificial ventilation/cooling	0	Other, Specify: ____________
		Passive ventilation	1	
		No ventilation	2	
8.	Bedding material	Yes	0	If yes, type of bedding: ____________
		No	2	
9.	Presence of sharp edges or items that cause injuries to the camel	No	0	
		Yes	2	
10.	Presence of other materials/items/goods being transported with camels in the vehicle	No	0	
		Yes	2	
11.	Presence of water/drinkers in the vehicle	Yes	0	If yes, type of type or number of drinkers: ____________
		No	2	
12.	Presence feed/feeder in the vehicle?	Yes	0	
		No	2	
13.	Number of camels in the truck	Numbers		
14.	Distance traveled	Km/miles		
15.	Number of stops during the journey	Number		
16.	Type of most of the roads along the journey route	Asphalt	0	Others: _______
		Gravel roads	1	
		Natural soil roads	2	
17.	Duration of the journey	Hours		

### 3.7 Assessment of the loading and unloading process

The types of loading and unloading are evaluated at departure and arrival locations, respectively. The checklist developed to evaluate loading and unloading is presented in Table 9.

**Table 9:** Checklist to evaluate the loading and unloading process agreed upon using EKEs.

Date: __________ ID of the Journey: __________________Ambient temperature: ________ Humidity: _________________				
No	Variables	Measurement scale/values	Score obtained	Remarks
Loading				
1.	Ambient temperature at the loading station	^0^C		
2.	Humidity at the loading station	%		
3.	Type of loading	Using ramp	0	Others: _______
		Manual	1	
		Machine lifting	2	
4.	Presence of a ramp for loading	Yes	0	
		No	2	
5.	Slope of the ramp at loading	Normal	0	
		Steep	2	
6.	Presence of lateral protection in the ramp at loading	Yes	0	
		No	2	
7.	Handling during loading	Gentle handling	0	Others: _______
		Use of excessive force	1	
		Restrained and pulled	2	
8.	Number of camels forced to load	Number		
9.	Number of camels self-loaded without being forced	Number		
10.	Number of camels which slip/fall during loading	Numbers		
11.	Vocalization during loading	No	0	If yes, how many? ________
		Yes	2	
12.	Duration of loading	Hours/Minutes		
Unloading				
13.	Ambient temperature at the unloading station	^0^C		
14.	Humidity at the unloading station			
15.	Type of unloading	Use of a ramp	0	Others: _______
		Manual lifting	1	
		Use of a lifting machine	2	
16.	Presence of a ramp for unloading	Yes	0	
		No	2	
17.	Slope of the ramp at unloading	Normal	0	
		Steep	2	
18.	Presence of lateral protection in the ramp	Yes	0	
		No	1	
19.	Handling during unloading	Gentle handling	0	
		Use of excessive force, such as pulling	1	
		Restrained and pulled, and dragged	2	
20.	Duration of unloading	Hours/min		
21.	Number of camels with slips/fells during unloading	Numbers		
22.	Number of camels forced to unload	Numbers		
23.	Number of camels self-unloaded without force			
24.	Vocalization during unloading	No	0	If yes, how many? ________
		Yes	2	
25.	The number of camels visibly sweating upon arrival	Numbers		
26.	Number of downers at arrival	Numbers		
27.	Number of camels dead on arrival	Numbers		
28.	Presence of diarrhea, blood, and excessive salivation in the cargo bed after unloading	No	0	
		Yes	2	

## 4 Discussion

This study aimed to develop a standardized welfare assessment protocol for the transport of dromedary camels through a systematic literature review and unstructured Expert Knowledge Elicitations (EKEs) (42). Usually, behavioral, physiological, and pathological parameters are used to assess the welfare of animals during transport (8, 13, 54). Assessing the welfare of farmed animals during transport helps identify sources of stress and improve handling practices. The outcome of the assessments will help to monitor welfare along the journey, and the assessors should be able to use the checklists and gather evidence which may be helpful in reducing the occurrence of transport-related injuries and health problems, enhancing productivity, and promoting humane treatment of animals during transportation. Additionally, welfare assessments help in developing better transport guidelines and standards, minimizing economic losses, and improving sustainable camel farming. To the authors’ knowledge, this is the first protocol developed to assess the transport condition of dromedary camels by road. This standard protocol might be useful to assess the welfare of dromedary camels during transport in several camel-rearing countries, and the findings will be an important asset in improving the transportation of dromedary camels worldwide.

The first checklist developed is crucial to assess the fitness for transport in dromedaries. Fitness for transport is one of the essential factors that determines the outcome of transportation (15, 55). There is no agreed-upon standard for determining whether a farm animal is fit for transport (15, 55). However, healthy, well-conditioned animals transported suffer fewer welfare consequences than those unfit for transport (1, 52, 55, 56). Ensuring that animals are free from parasitic disease (54) and fit for transport has paramount importance for their welfare during transport (11, 52, 53, 57). The criteria to determine whether an animal is fit for transport vary across different species. The fitness for transportation should be assessed by listing conditions that make the animal unable to cope with transportation stress, leading to exacerbation of the condition, leading to bad outcomes (15). Although there is no literature or relevant studies on the fitness of transporting dromedary camels, there are studies on other species, such as cattle, pigs, and horses. A 10-year record file study in Denmark (58) showed that wounds, umbilical or inguinal outpouching, lameness, prolapse, and illness in pigs, as well as lameness, wounds, prolapse, and illness in cattle, were conditions that made them unfit for transport in ascending order. A 2022 European Union Food Safety Authority (EFSA) scientific opinion lists sickness (impaired health), injury, experiencing pain, emaciation (poor body conditions), weakness, fatigue/exhaustion, dehydration, engorged udder, prolapse, hernia, lameness, pregnancy, and recent calving/lambing as conditions rendering cattle, sheep, and goats unfit for transport (14, 15). Since there are no specific lists for dromedary camels, the above-listed conditions are considered criteria in the protocol for determining their fitness for transport.

The second checklist proposed in our protocol should be filled in after loading, but loading should also be observed and data recorded. It is necessary to evaluate dromedary camels after loading, to evaluate whether they sustained some kind of injury, observe their behavior during the loading process, and determine whether they are still fit for transport. Furthermore, after loading, the loading density should be calculated and recorded. Loading is one of the most stressful processes in the transport of farmed animals; animals are often highly stressed and may be exposed to hazards that compromise their welfare and health (53). Specifically, how the animals are handled, the use and slope of the ramp are some of the factors that determine the quality of loading (9, 53). Rough handling during loading, mixing unfamiliar animals, and the type of loading trigger stress in animals during transportation (9, 53, 59). Consequently, the World Organization for Animal Health (WOAH) suggests using stress-free handling methods EndNote (41), and for camels, a new method using positive reinforcement has been developed (60) and should be implemented. The loading density affects animal behavior during transportation due to restricted space, unwanted contact, and poor visibility, resulting in behavioral changes (61). Whether the space provided to the animals in the vehicle is too little or too large, it can alter the health and welfare of animals (12). In a study conducted in Morocco, Lemrhamed et al. (62) stated that increasing the loading density by reducing the space allowance from 2 to 3.6 m^2^ per camel to 1.44–1.80 m^2^ per camel altered the physiological parameters of dromedary camels and affected the quality of meat. Therefore, space allowance is an important factor in dromedary camel transportation, and future studies should be conducted to determine the space requirement of dromedary camels during vehicle transportation.

The third proposed checklist is during the journey. Animals are exposed to different factors that negatively affect their welfare and health during transportation; these could result from the movement of trucks, adverse weather conditions, noise, and mixing of unfamiliar animals during the journey (9, 15, 63). Animals could show increased abnormal behaviors during the journey, which could be attributed to different factors (61). Therefore, to evaluate the condition of the dromedary camels, their welfare should be assessed regularly during the journeys, at least at each drive’s rest stop. In addition, the posture of animals during transportation should be recorded and evaluated to determine their welfare. During transportation in a vehicle, small animals such as sheep, poultry, and pigs prefer to lie down, but they may not get sufficient space for rest, while large animals such as cattle rarely lie down, even though they need recumbent rest (13). Often animals decide to not lie down, as vehicle movement during transportation triggers a stress response making them restless (12). During transport vehicle vibration, braking, and the floor conditions make animals uncomfortable and affect the ability of animals to keep their balance t and impair their ability to lie down and rest (13). The presence of bedding material is the other factor that determines the preference of animals to rest during transportation. Olivares Guzmán et al. (64) reported that the presence of bedding increased the likelihood of cattle lying down sooner and eating later at rest stops, suggesting that bedding may help reduce fatigue during long-distance transport. If the journey duration is long, it is necessary to ensure the floor conditions are comfortable and there is enough space so that the animals can rest (13). Additionally, bedding material can prevent injury to the animal (e.g., a hematoma), especially if the road is bumpy (65). Monitoring the proportion of animals able to rest, the space allowance and the conditions of the bedding during the journey will be crucial indicators to understand the level of welfare of the transported animals.

The fourth and fifth proposed checklists are the evaluation of the dromedary camels upon arrival and 1 h after arrival. Upon arrival and 1 h after arrival, camels should be evaluated to assess their welfare, determine stress levels, identify the presence of injuries, and evaluate their behavioral responses. Two different checklists were proposed, in line with the literature performed on other species (35, 45), as immediately after transport, it is well known that animals may still be affected by the long restraint and fasting and other transport-related stressors (14), and show abnormal behavioral, physiological and clinical indicators. Most of those indicators should go back to normal 1 h after traveling, and if not, the animals should be treated by a veterinarian and declared no fit to be slaughtered or continue to travel (44). The journey duration, handling at unloading, the experience of the drivers and the handlers, the design of the vehicle, and stops during the journey affect the welfare of animals during transportation and determine their welfare at arrival (9, 35, 53, 66). The duration of the journey, unloading periods, and the unloading environment temperature affect the physiological parameters of animals (3). Downers and mortality are important indicators of animal welfare during transportation (9, 67, 68). Some factors that predispose animals to become non-ambulatory after transportation include the duration of the journey, ambient temperature, and poor physical condition (68). The death of animals may occur after transportation, which is an indirect indicator of the welfare of the journey conditions (67, 69). Malena et al. (67) reported that the highest mortality rates were in young sows and boars (0.2562%), followed by fattening pigs (0.1075%), dairy cows (0.0396%), calves (0.0269%), and fattening cattle (0.0069%). Factors such as age, species, fattened or not, poor physical conditions, and fitness for transport affect the mortality that occurs during transportation (67, 69). Therefore, the welfare of camels should be assessed immediately after unloading and 1 h after arrival to evaluate their mental and physical state and recovery from transport stress. Furthermore, the number of downers at arrival and 1 h after arrival should be recorded, as downers are animals that should be euthanised in the vehicle. Currently, there are no studies, and consequently, data on mortality rate or occurrence of downers in dromedary camels, so the proposed check lists will be useful to gather evidence, which could lead to policy changes.

The sixth checklist proposed in our protocol is to evaluate the journey and the vehicle. Structures and facilities that may have a potentially hazardous impact on the welfare of the animals during transport should be evaluated, such as the vehicle design, the presence of ventilation, a loading and unloading facility, and space allowance within the vehicle, which determine the welfare of animals being transported (9, 70). The truck should be evaluated for its dimensions to determine the space allowance, the presence of an appropriate ramp for loading the camels, the availability of bedding material, the absence of sharp edges, the presence of ventilation, and the presence of roofs to protect the camels from extreme weather (12, 36). Transport duration, the roads used, transport temperature, and stops during the journey affect the welfare of the animals being transported (12, 13). Therefore, the journey conditions should also be evaluated. Dromedary camels may not be susceptible to harsh hot temperatures and can resist them (18). However, the temperature of the vehicles during transportation is an important factor that affects the welfare of animals being transported (16, 71). Due to the difficulty of measuring vehicle temperature during the journey in field conditions, we only included environmental temperature at departure and destination in our protocols as indicators.

The final checklist in the proposed protocol contains an indicator to evaluate loading and unloading processes. Loading and unloading are also one of the most stressful processes of animal transportation (9, 72). The handling, the structures, or equipment used during unloading determine the welfare of animals (9, 36). Miranda et al. (36) in their study of welfare assessment during unloading of horses stated that there is a correlation between ramp slope and falling, the type of ramp floor and slipping, fast gait, and the presence of gaps between the ramp and the floor. Therefore, it is necessary to evaluate the handling of animals, the availability of the ramp, the slope of the ramp, the durations, falls, slips, and reluctance during the unloading process (9, 36). In addition, the environmental temperature and humidity at loading (departure) and unloading (destination) stations should be recorded. A study by Padalino et al. (73) on beef cattle transported from France to Italy found that sudden temperature changes between departure and arrival were predisposing factors for the occurrence of bovine respiratory syndrome after traveling.

Like other theoretical protocols, this protocol has certain limitations (38, 39). The first limitation is that most of the proposed indicators in the checklists have not yet been validated under practical field conditions because only a small number of observations of dromedary camel transportation were used to pilot the presented protocol. It would be useful in future studies to assess cortisol in saliva to validate the proposed welfare-indicators and compare the overall welfare estimated with the protocol with the cortisol levels. Moreover, the animals we used for the pilot were mainly young males going to slaughter. Therefore, the proposed checklists should be applied in several data collections, including all genders and possible physiological states, to be refined and validated. Another limitation is the scarcity of scientific literature addressing the welfare of dromedary camels specifically during transportation. Due to the lack of species-specific information, the protocol was based partly on welfare assessment tools developed for other livestock species transported under different conditions. While these studies provided useful guidance, differences in species behavior, physiology, and management systems may affect the relevance of certain indicators. Consequently, further validation studies are necessary to test the protocol under field conditions. The protocol may require refinement and modification once it is applied to different transportation scenarios and management systems. However, notwithstanding the above-mentioned limitation, this is the first protocol tailored to assess the welfare of dromedary camels during transport and having a standard protocol which checklists are published and freely available may help in a harmonized data collection, providing the evidence needed to propose legislation and guidelines on the protection of the welfare of dromedary camels. Policymakers require robust evidence to inform legislation aimed at protecting animal welfare during transport; therefore, applying this protocol in camel-rearing countries will generate the data needed to support the development of evidence-based policies.

## 5 Conclusion

This study describes for the first time a protocol to assess the welfare of dromedary camels during road transport. Several animal-based and environmental welfare indicators were included in the checklists to evaluate the camels at different stages of transportation, namely the assessment of the camel before loading, after loading, during the journey, upon arrival, and 1 h after arrival; the protocol also includes an *ad hoc* checklist to assess the loading and unloading process, the vehicle, and the journey. The protocol was developed using a literature search and experiential knowledge elicitation (EKE), aiming to use non-invasive methods and feasible indicators. However, it was piloted with only a few journeys and camels, so further studies are needed to apply it properly and refine and validate the proposed measurement indicators. Future studies should also focus on integrating monitoring technologies (e.g., GPS, sensors) in future iterations of the protocol for more precise welfare assessment.

## Data Availability

The data analyzed in this study is subject to the following licenses/restrictions: the data can be requested to the authors. Requests to access these datasets should be directed to barbara.padalino@scu.edu.au.
